# Risk factors for hospitalization among persons with COVID-19—Colorado

**DOI:** 10.1371/journal.pone.0256917

**Published:** 2021-09-02

**Authors:** Grace M. Vahey, Emily McDonald, Kristen Marshall, Stacey W. Martin, Helen Chun, Rachel Herlihy, Jacqueline E. Tate, Breanna Kawasaki, Claire M. Midgley, Nisha Alden, Marie E. Killerby, J. Erin Staples, on behalf of the Colorado Investigation Team

**Affiliations:** 1 Centers for Disease Control and Prevention, Atlanta, Georgia, United States of America; 2 Epidemic Intelligence Service, Centers for Disease Control and Prevention, Atlanta, Georgia, United States of America; 3 Colorado Department of Public Health and Environment, Denver, Colorado, United States of America; University "Magna Graecia" of Catanzaro, ITALY

## Abstract

**Background:**

Most current evidence on risk factors for hospitalization because of coronavirus disease 2019 (COVID-19) comes from studies using data abstracted primarily from electronic health records, limited to specific populations, or that fail to capture over-the-counter medications and adjust for potential confounding factors. Properly understanding risk factors for hospitalization will help improve clinical management and facilitate targeted prevention messaging and forecasting and prioritization of clinical and public health resource needs.

**Objectives:**

To identify risk factors for hospitalization using patient questionnaires and chart abstraction.

**Methods:**

We randomly selected 600 of 1,738 laboratory-confirmed Colorado COVID-19 cases with known hospitalization status and illness onset during March 9–31, 2020. In April 2020, we collected demographics, social history, and medications taken in the 30 days before illness onset via telephone questionnaire and collected underlying medical conditions in patient questionnaires and medical record abstraction.

**Results:**

Overall, 364 patients participated; 128 were hospitalized and 236 were non-hospitalized. In multivariable analysis, chronic hypoxemic respiratory failure with oxygen requirement (adjusted odds ratio [aOR] 14.64; 95% confidence interval [CI] 1.45–147.93), taking opioids (aOR 8.05; CI 1.16–55.77), metabolic syndrome (aOR 5.71; CI 1.18–27.54), obesity (aOR 3.35; CI 1.58–7.09), age ≥65 years (aOR 3.22; CI 1.20–7.97), hypertension (aOR 3.14; CI 1.47–6.71), arrhythmia (aOR 2.95; CI 1.00–8.68), and male sex (aOR 2.65; CI 1.44–4.88), were significantly associated with hospitalization.

**Conclusion:**

We identified patient characteristics, medications, and medical conditions, including some novel ones, associated with hospitalization. These data can be used to inform clinical and public health resource needs.

## Introduction

Since the first cases of coronavirus disease 2019 (COVID-19), the disease caused by severe acute respiratory syndrome coronavirus 2 (SARS-CoV-2), were reported from China in late December 2019, the subsequent pandemic has resulted in millions of cases worldwide, including over 33.9 million cases and 600,000 deaths in the United States as of July 18, 2021 [1]. Early descriptions of hospitalized COVID-19 patients from China, Italy, and the United States found that large proportions of patients had underlying medical conditions [2–6]. As the pandemic progressed, many underlying medical conditions have been implicated as potential risk factors for severe COVID-19 illness (e.g., hospitalization, intensive care unit [ICU] admission, intubation, death) including cardiovascular disease, chronic kidney disease, chronic respiratory disease, diabetes mellitus (DM), hypertension, and obesity [7–13]. Patient characteristics, specifically older age, male sex, certain racial or ethnic groups, and smoking history had also been associated with increased risk of severe COVID-19 [11, 12, 14–16]. Additionally, early in the pandemic it was proposed that certain medications, including angiotensin-converting enzyme inhibitors (ACE inhibitors), angiotensin-receptor blockers (ARBs), and non-steroidal anti-inflammatory drugs (NSAIDs), could enhance SARS-CoV-2 binding and thus increase pathogenicity [17–20]. However, more recent reports have not found this association [21–25].

Early in the pandemic, most evidence on risk factors for hospitalization due to COVID-19 came from retrospective cohort studies and case series that used data abstracted solely from electronic health records [7, 14, 26–35], were limited to specific populations and types of data collected [30–32, 36–42], or failed to adjust for potential confounding factors such as age, sex, or other comorbidities [2, 34, 35, 43]. An improved understanding of factors driving healthcare utilization will inform clinical and public health guidance, facilitate messaging to high-risk groups, and allow for better estimates of clinical and public health resource needs, including preventive (i.e., vaccines), diagnostic, and therapeutic resource allocations. In this case-control study, we use data from interviews and medical record review to identify patient characteristics, underlying medical conditions, and medications that increase the risk of hospitalization among persons with laboratory-confirmed COVID-19.

## Methods

### Sample

Hospitalized and non-hospitalized patients were identified from laboratory-confirmed COVID-19 cases reported to the Colorado Electronic Disease Reporting System (CEDRS) as of April 5, 2020. Based on data available in CEDRS, patients were considered eligible if they resided in one of nine contiguous counties accounting for ~80% of Colorado’s population (Adams, Arapahoe, Boulder, Denver, Douglas, El Paso, Jefferson, Larimer, and Weld), had known hospitalization status, and self-reported illness onset during March 9–31, 2020 when there was ongoing community transmission and wider availability of SARS-CoV-2 testing in Colorado. To obtain at least 300 patient interviews, including 200 non-hospitalized and 100 hospitalized, we stratified by hospitalization status and used simple random sampling to select 600 patients from 1,738 COVID-19 cases meeting inclusion criteria.

### Data collection

At least three attempts were made to contact selected patients on at least two separate days at different times of day during April 10–30, 2020. A standardized questionnaire was administered by telephone to patients who agreed to participate by providing oral consent. Demographic information, social history, underlying medical conditions, and medications and supplements taken in the 30 days prior to illness onset were obtained and hospitalization status and illness onset date from CEDRS were verified. Proxy (e.g., relative or caregiver) interviews were carried out for deceased patients, minors, and persons unable to be interviewed (e.g., those with dementia). Once an interview was complete, medical record abstraction was performed for all patients with records related to their COVID-19 illness available in three electronic medical record repositories covering the major medical systems in the selected counties. A standardized medical record abstraction form was used to collect information on underlying medical conditions and course of illness.

This activity was reviewed by the Centers for Disease Control and Prevention’s (CDC) Human Research Protection Office and determined to be exempt from human participants’ research regulations, including the need for documented written consent, as the activities involved identification, control or prevention of disease in response to an immediate public health threat. It was conducted consistent with applicable federal law and CDC policy (See e.g., 45 C.F.R. part 46, 21 C.F.R. part 56; 42 U.S.C. §241(d); 5 U.S.C. §552a; 44 U.S.C. §3501 et seq.).

### Statistical analysis

Data were entered into a Research Electronic Data Capture database [44, 45]. Prescribed and over-the-counter (OTC) medications and supplements taken in the 30 days prior to illness onset were collected as free text during interviews and were categorized into general drug classes by three clinicians (EM, HC, and ES). Participants were considered to have an underlying medical condition if it was reported in either their interview or medical record as the latter has been shown to be a more comprehensive approach to capture these data [46]. Body Mass Index (BMI) was calculated in kg/m^2^ using height and weight reported in interviews.

Frequencies and percentages were calculated stratified by hospitalization status. Univariable logistic regression was performed to investigate the association of individual patient characteristics, underlying medical conditions, and medications with the outcome of hospitalization; crude odds ratios (OR) and 95% confidence intervals (CI) were calculated. A multivariable logistic regression model for hospitalization was performed to calculate adjusted ORs (aOR) for factors previously reported to be associated with hospitalization or severe disease including age, sex, race, ethnicity, insurance status, smoking status, alcohol use, BMI, hypertension, DM, cardiovascular disease (excluding hypertension), chronic renal disease, and chronic respiratory disease [7–12]. A series of multivariable logistic regression models were then conducted to calculate aORs for individual patient characteristics, medications, and underlying medical conditions adjusted for the previously reported risk factors listed above. When evaluating the association of individual medical conditions of interest within organ system or disease categories with hospitalization, the disease category variable excluded the individual medical condition being evaluated as a risk factor. For example, when calculating the aOR for asthma, the chronic respiratory disease variable included all chronic respiratory diseases except asthma. We assessed collinearity among all variables that were adjusted for in multivariable analysis; no significant collinearity was identified. Univariable and multivariable analyses were only performed for variables reported by 10 or more patients. Statistical analyses were conducted using SAS 9.4 (SAS Institute Cary, NC) and R version 3.6.3 [47].

## Results

Of 600 randomly selected patients, 364 (61%) completed the interview, 46 (8%) were ineligible (i.e., illness onset date prior to March 9 or asymptomatic), 57 (10%) declined to participate, and 133 (22%) were unreachable. Median age of the 364 participating patients was 50 years (range 2 months–94 years), 187 (51%) were male, 279 (77%) identified as White, and 75 (21%) as Hispanic. Almost all (345; 95%) reported having health insurance, and 128 (35%) were hospitalized. Eighteen (5%) patients died, including 15 who were hospitalized and 3 who were not. Compared with patients who declined to participate or were unreachable, participating patients resided proportionately in the same counties, and had similar hospitalization rates (35% versus 31%) and case-fatality ratios (5% vs 8%) but were older than non-participating patients (median age 50 vs 43 years).

Hospitalized patients were older than non-hospitalized patients, with median ages of 61 years (interquartile range [IQR] 48–72 years) and 44 years (IQR 31–57 years), respectively. On univariable analysis when compared to non-hospitalized patients, hospitalized patients more frequently reported being male, having only public health insurance, and having a history of smoking (Table 1). Hospitalized patients less frequently reported current marijuana use or any alcohol consumption within the last year when compared to non-hospitalized patients. Hospitalization status did not differ by race or ethnicity.

**Table 1:** Demographic characteristics and social behaviors reported in interview among persons with laboratory-confirmed COVID-19, by hospitalization status (n = 364)—Colorado, March 2020.

		Hospitalized (n = 128)		Non-hospitalized (n = 236)	Crude OR^1^ (95%CI)		Adjusted OR^2^ (95%CI)
		**n (%)**		**n (%)**			
**Age group, *y***							
	<18	3 (2)		1 (0)	--		--
	19–44	23 (18)		118 (50)	Reference		Reference
	45–64	50 (39)		84 (36)	**3.05 (1.73–5.39)**		1.97 (0.99–3.95)
	≥65	52 (41)		33 (14)	**8.08 (4.33–15.09)**		**3.22 (1.20–7.97)**
**Sex**							
	Male	79 (62)		108 (46)	**1.91 (1.23–2.96)**		**2.65 (1.44–4.88)**
	Female	49 (38)		127 (54)	Reference		Reference
	Other	0 (0)		1 (0)	--		--
**Race**							
	White	90 (70)		189 (80)	Reference		Reference
	Black	13 (10)		12 (5)	2.28 (0.99–5.19)		1.26 (0.39–4.02)
	Asian^3^	8 (6)		8 (3)	2.1 (0.76–5.78)		--
	Pacific Islander^3^	1 (1)		1 (0)	--		--
	American Indian^3^	2 (2)		1 (0)	--		--
	Other^3^	5 (4)		17 (7)	0.62 (0.22–1.73)		1.22 (0.55–2.69)
	Unknown	4 (3)		4 (2)	--		--
	Multiracial^3^	5 (4)		4 (2)	--		--
**Ethnicity**							
	Non-Hispanic	86 (67)		163 (69)	Reference		Reference
	Hispanic	29 (23)		46 (19)	1.2 (0.70–2.04)		1.1 (0.53–2.31)
	Unknown	13 (10)		27 (11)	0.91 (0.45–1.86)		0.69 (0.24–1.96)
**Health insurance**							
	Private Insurance	68 (53)		198 (84)	Reference		Reference
	Public Insurance^4^	50 (39)		29 (12)	**5.02 (2.94–8.56)**		1.8 (0.81–4.00)
	Uninsured	8 (6)		6 (3)	**3.88 (1.30–11.59)**		3.43 (0.80–14.71)
	Unknown	2 (2)		3 (1)	--		--
**Smoking history**							
Ever Smoker		60 (47)		67 (28)	**2.23 (1.42–3.48)**		1.31 (0.72–2.38)
Current Smoker		3 (2)		5 (2)	--		--
Currently Vape		3 (2)		9 (4)	0.61 (0.16–2.28)		0.4 (0.05–2.99)
**Current recreational drug use**							
Marijuana (THC)		6 (5)		32 (14)	**0.31 (0.13–0.77)**		1.49 (0.47–4.75)
	Inhale	4 (3)		18 (8)	0.39 (0.13–1.18)		1.48 (0.35–6.27)
	Consume	3 (2)		22 (9)	**0.23 (0.07–0.80)**		1.64 (0.39–6.97)
Cocaine		0 (0)		2 (1)	--		--
Methamphetamine		1 (1)		0 (0)	--		--
Heroin		0 (0)		0 (0)	--		--
**Alcohol consumption in the past year**							
	Never	48 (38)		39 (17)	Reference		Reference
	≤ Once per month	31 (24)		53 (22)	**0.48 (0.26–0.88)**		0.92 (0.40–2.09)
	2–4 times per month	27 (21)		53 (22)	**0.41 (0.22–0.78)**		1.37 (0.57–3.32)
	2–3 times per week	10 (8)		52 (22)	**0.16 (0.07–0.35)**		0.42 (0.14–1.23)
	≥4 times per week	12 (9)		39 (17)	**0.25 (0.12–0.54)**		0.68 (0.23–1.97)

Abbreviations: CI–confidence interval; OR–odds ratio; y–years.

^1^Exact methods were used in crude analysis if there was one or more expected cell count less than 5.

^2^Multivariable model used for adjustment included age, sex, race, ethnicity, insurance status, smoking history, alcohol use, BMI, hypertension, diabetes, cardiovascular disease, chronic renal disease, and chronic respiratory disease.

^3^For multivariable analysis, all races except White or Black were combined into one category.

^4^Reported only having Medicaid or Medicare.

Medications that were reportedly taken in the 30 days prior to illness onset among hospitalized patients and non-hospitalized patients are shown in Table 2. Anticoagulants, antihyperglycemics, antihypertensives, cholesterol medications, neuropathic pain treatments, opioids, and pain/fever reducing medications were significantly associated with hospitalization in crude univariable analysis.

**Table 2:** Medications reported in interview to have been taken by persons with laboratory-confirmed COVID-19 in the 30 days prior to illness onset, by hospitalization status (n = 364)—Colorado, March 2020.

Medication category		Hospitalized (n = 128)	Non-hospitalized (n = 236)	Crude OR^1^ (95%CI)	Adjusted OR^2^ (95%CI)
		**n (%)**	**n (%)**		
Allergy and antihistamine		13 (10)	19 (8)	1.29 (0.62–2.71)	1.01 (0.37–2.75)
Antacid		13 (10)	13 (6)	1.93 (0.87–4.32)	0.65 (0.21–2.01)
Antiarrhythmic		2 (2)	0 (0)	--	--
Antibiotic		1 (1)	5 (2)	--	--
Anticoagulant/antiplatelet		25 (20)	14 (6)	**3.85 (1.92–7.71)**	1.04 (0.40–2.65)
Antiemetic		0 (0)	0 (0)	--	--
Antiepileptic		3 (2)	1 (0)	--	--
Antihyperglycemic		21 (16)	17 (7)	**2.53 (1.28–4.99)**	0.8 (0.22–2.84)
Antihypertensives		52 (41)	27 (11)	**5.29 (3.11–9.03)**	0.95 (0.37–2.44)
	ACE inhibitor	19 (15)	8 (3)	**4.97 (2.11–11.70)**	0.8 (0.27–2.37)
	Angiotensin receptor blocker	14 (11)	6 (3)	**4.71 (1.76–12.57)**	1.5 (0.40–5.62)
	Beta blocker	19 (15)	8 (3)	**4.96 (2.11–11.70)**	1.24 (0.42–3.69)
	Calcium channel blocker	15 (12)	8 (3)	**3.78 (1.56–9.19)**	1.02 (0.32–3.22)
	Thiazide	6 (5)	5 (2)	2.27 (0.68–7.60)	0.58 (0.13–2.52)
	Other diuretic	11 (9)	4 (2)	**5.45 (1.70–17.50)**	2.57 (0.53–12.49)
	Other	1 (1)	0 (0)	--	--
Antitussive		1 (1)	2 (1)	--	--
Antiviral		0 (0)	6 (3)	--	--
Asthma treatment (oral)		6 (5)	6 (3)	1.89 (0.60–5.97)	1.02 (0.21–4.92)
Cancer treatment		5 (4)	1 (0)	--	--
Cholesterol treatment		28 (22)	23 (10)	**2.59 (1.42–4.73)**	0.81 (0.34–1.96)
Decongestant		1 (1)	5 (2)	--	--
Hormone replacement		2 (2)	5 (2)	--	--
Hypothyroid treatment		10 (8)	12 (5)	1.58 (0.66–3.77)	0.57 (0.17–1.94)
Immunosuppressant		1 (1)	1 (0)	--	--
Inhaler (including inhaled steroids)		16 (13)	19 (8)	1.63 (0.81–3.30)	0.83 (0.29–2.42)
Mental health treatment		25 (20)	36 (15)	1.34 (0.77–2.37)	1.41 (0.65–3.08)
Migraine treatment		0 (0)	5 (2)	--	--
Muscle relaxant		6 (5)	3 (1)	--	--
Neuropathic pain treatment		10 (8)	3 (1)	**6.58 (1.78–24.37)**	1.31 (0.26–6.68)
Nitrates (cardiac)		2 (2)	0 (0)	--	--
NSAID		27 (21)	48 (20)	1.05 (0.62–1.78)	0.51 (0.25–1.05)
Opioid		8 (6)	2 (1)	**7.8 (1.63–37.31)**	**8.05 (1.16–55.77)**
Oral Contraceptive		0 (0)	14 (6)	--	--
Pain medication/fever reducer		26 (20)	26 (11)	**2.06 (1.14–3.72)**	1.63 (0.71–3.70)
Phosphodiesterase-5 enzyme inhibitor		0 (0)	0 (0)	--	--
Steroids		4 (3)	3 (1)	--	--
	Topical	0 (0)	1 (0)	--	--
Vitamins and supplements		54 (42)	107 (45)	0.88 (0.57–1.36)	0.72 (0.40–1.30)

Abbreviations: CI–confidence interval; OR–odds ratio.

^1^Exact methods were used in crude analysis if there was one or more expected cell count less than 5.

^2^Multivariable model used for adjustment included age, sex, race, ethnicity, insurance status, smoking history, alcohol use, BMI, hypertension, diabetes, cardiovascular disease, chronic renal disease, and chronic respiratory disease.

Hospitalized patients had a higher median BMI (30; IQR 26–35) than non-hospitalized patients (26; IQR 23–30) and reported more individual underlying medical conditions among all organ systems and disease categories when compared with non-hospitalized patients (Table 3). Chronic lung disease, cardiovascular disease, endocrine disorders, renal disease, liver disease, autoimmune disorders, hematologic disorders, cancer, neurologic or neurodevelopmental disorders, and psychiatric diagnoses were all significantly associated with hospitalization in crude univariable analysis. Immunocompromising conditions were the only broad category not associated with hospitalization status on univariable analysis.

**Table 3:** Underlying medical conditions reported in interviews or medical records among persons with laboratory-confirmed COVID-19, by hospitalization status (n = 364)—Colorado, March 2020.

			Hospitalized (n = 128)	Non-hospitalized (n = 236)	Crude OR^1^ (95%CI)	Adjusted OR^2^ (95%CI)
			**n (%)**	**n (%)**		
**Cardiovascular disease**			75 (59)	49 (21)	**5.4 (3.37–8.66)**	1.11 (0.51–2.43)
	Hypertension		68 (53)	29 (12)	**8.09 (4.80–13.62)**	**3.14 (1.47–6.71)**
	Coronary artery disease, heart attack		23 (18)	4 (2)	**12.7 (4.29–37.66)**	3.33 (0.87–12.77)
	Heart failure, congestive heart failure		9 (7)	1 (0)	**17.77 (2.23–141.94)**	2.47 (0.25–24.85)
	Cerebrovascular accident, stroke		3 (2)	3 (1)	--	--
	Congenital heart disease		0 (0)	1 (0)	--	--
	Valvular heart disease		2 (2)	1 (0)	--	--
	Arrhythmia		17 (13)	10 (4)	**3.46 (1.53–7.81)**	**2.95 (1.00–8.68)**
	Hyperlipidemia^3^		27 (21)	8 (3)	--	--
**Chronic lung disease**			56 (44)	51 (22)	**2.82 (1.77–4.50)**	1.82 (0.97–3.39)
	Asthma or reactive airway disease		24 (19)	36 (15)	1.28 (0.73–2.26)	1.17 (0.54–2.54)
	Emphysema, COPD, or chronic bronchitis		18 (14)	6 (3)	**6.27 (2.42–16.24)**	3.01 (0.80–11.31)
	Interstitial lung disease		0 (0)	0 (0)	--	--
	Pulmonary fibrosis		1 (1)	0 (0)	--	--
	Restrictive lung disease		3 (2)	1 (0)	--	--
	Sarcoidosis		0 (0)	0 (0)	--	--
	Cystic fibrosis		0 (0)	0 (0)	--	--
	Chronic hypoxemic respiratory failure with oxygen requirement		13 (10)	1 (0)	**26.57 (3.43–205.56)**	**14.64 (1.45–147.93)**
	Obstructive sleep apnea		18 (14)	10 (4)	**3.7 (1.65–8.28)**	0.67 (0.23–1.97)
	Active tuberculosis		1 (1)	2 (1)	--	--
**Endocrine disorder**			65 (51)	54 (23)	**3.48 (2.19–5.51)**	1.81 (0.91–3.59)
	Diabetes Mellitus		34 (27)	20 (8)	**3.9 (2.14–7.14)**	1.08 (0.45–2.61)
	Pre-diabetes		10 (8)	13 (6)	1.45 (0.62–3.42)	0.9 (0.31–2.57)
	Hypothyroidism^3^		18 (14)	15 (6)	--	--
**Renal disease**			25 (20)	11 (5)	**4.96 (2.35–10.47)**	1.71 (0.62–4.70)
	Chronic kidney disease or insufficiency		13 (10)	5 (2)	**5.22 (1.82–15.00)**	0.66 (0.15–2.91)
	End-stage renal disease		5 (4)	0 (0)	--	--
	Dialysis		4 (3)	0 (0)	--	--
		Hemodialysis	3 (2)	0 (0)	--	--
		Peritoneal	1 (1)	0 (0)	--	--
**Liver disease**			9 (7)	4 (2)	**4.39 (1.32–14.54)**	2.21 (0.49–10.04)
	Alcoholic hepatitis		0 (0)	0 (0)	--	--
	Chronic liver disease		0 (0)	0 (0)	--	--
	Cirrhosis or end stage liver disease		1 (1)	0 (0)	--	--
	Hepatitis B, chronic		1 (1)	0 (0)	--	--
	Hepatitis C, chronic		1 (1)	0 (0)	--	--
	Non-alcoholic fatty liver disease		2 (2)	3 (1)	--	--
**Autoimmune disorder**			13 (10)	11 (5)	**2.31 (1.00–5.32)**	2.58 (0.82–8.16)
	Rheumatoid arthritis		4 (3)	2 (1)	--	--
	Systemic lupus		1 (1)	1 (0)	--	--
**Hematologic disorder**			21 (16)	14 (6)	**3.11 (1.52–6.36)**	2.18 (0.88–5.43)
	Anemia		13 (10)	7 (3)	**3.7 (1.43–9.52)**	2.04 (0.56–7.44)
	Sickle cell disease		0 (0)	0 (0)	--	--
	Sickle cell trait		0 (0)	0 (0)	--	--
	Bleeding or clotting disorder		5 (4)	2 (1)	--	--
**Immunocompromised condition**			9 (7)	10 (4)	1.71 (0.68–4.32)	1.42 (0.39–5.13)
	HIV infection		0 (0)	1 (0)	--	--
	AIDS		0 (0)	0 (0)	--	--
	Solid organ transplant		3 (2)	0 (0)	--	--
	Stem cell transplant		0 (0)	0 (0)	--	--
	Leukemia		1 (1)	1 (0)	--	--
	Lymphoma		0 (0)	1 (0)	--	--
	Multiple myeloma		1 (1)	0 (0)	--	--
	Splenectomy or asplenia		0 (0)	2 (1)	--	--
**Cancer**			22 (17)	18 (8)	**2.51 (1.29–4.89)**	1.6 (0.66–3.86)
	IV chemotherapy		6 (5)	5 (2)	2.27 (0.68–7.60)	2.79 (0.57–13.65)
	Oral chemotherapy		1 (1)	2 (1)	--	--
	Radiation		6 (5)	6 (3)	1.89 (0.60–5.97)	1.51 (0.36–6.35)
	Other		12 (9)	9 (4)	**2.61 (1.07–6.37)**	1.86 (0.55–6.31)
**Neurologic or neurodevelopmental disorder**			27 (21)	16 (7)	**3.68 (1.90–7.12)**	1.61 (0.66–3.92)
	Migraines^3^		3 (2)	11 (5)	--	--
	Dementia^3^		4 (3)	5 (2)	--	--
**Psychiatric diagnosis**			40 (31)	49 (21)	**1.73 (1.06–2.83)**	1.17 (0.58–2.36)
	Depression^3^		27 (21)	30 (13)	--	--
	Anxiety^3^		21 (16)	32 (14)	--	--
**Other chronic diseases**			78 (61)	51 (22)	--	--
	Gastroesophageal reflux disease^3^		20 (16)	5 (2)	--	--
	Allergic rhinitis^3^		8 (6)	11 (5)	--	--
	Arthritis^3^		14 (11)	5 (2)	--	--
	Chronic pain^3^		7 (5)	1 (0)	--	--
	Benign prostatic hyperplasia^3^		7 (5)	0 (0)	--	--
	Bone density abnormality^3^		2 (2)	4 (2)	--	--
**BMI** ^**4**^ **(kg/m** ^**2**^ **)**						
	Underweight (<18.5)		3 (2)	1 (0)	--	--
	Normal (18.5 to <25)		23 (18)	93 (39)	Reference	Reference
	Overweight (25 to <30)		31 (24)	85 (36)	1.48 (0.80–2.73)	0.66 (0.29–1.49)
	Obese (30+)		67 (52)	54 (23)	**5.02 (2.81–8.96)**	**3.35 (1.58–7.09)**
		Class 1 (30 to <35)	38 (30)	34 (14)	**4.52 (2.36–8.66)**	--
		Class 2 (35 to <40)	20 (16)	15 (6)	**5.39 (2.40–12.12)**	--
		Class 3 (40+)	9 (7)	5 (2)	**7.28 (2.23–23.80)**	--
	Unknown		4 (3)	3 (1)	--	--
**Metabolic Syndrome** ^5^			19 (15)	2 (1)	**20.39 (4.67–89.11)**	**5.71 (1.18–27.54)**

Abbreviations: CI–confidence interval; OR–odds ratio.

^1^Exact methods were used in crude analysis if there was one or more expected cell count less than 5.

^2^Multivariable model used for adjustment included age, sex, race, ethnicity, insurance status, ever smoking, alcohol use, BMI, hypertension, diabetes, cardiovascular disease, chronic renal disease, and chronic respiratory disease.

^3^These variables were categorized from free text responses to “Other” chronic disease answers. Because they were not collected systematically like the other underlying medical conditions, descriptive statistics alone are reported.

^4^Body mass index (BMI) was calculated from height and weight reported during interviews.

^5^Multivariable model when investigating metabolic syndrome did not include individual hypertension, diabetes, and BMI variables.

In multivariable analysis, age ≥65 years (aOR 3.22; 95% CI 1.20–7.97) and male sex (aOR 2.65; 95% CI 1.44–4.88) were the only patient characteristics significantly associated with hospitalization (Table 1). Additionally, history of taking opioids (aOR 8.05; 95% CI 1.16–55.77) was significantly associated with hospitalization. Opioids that patients noted taking in the 30 days before their illness onset included buprenorphine, hydrocodone, hydromorphone, morphine, oxycodone, and tramadol. Among underlying medical conditions, chronic hypoxemic respiratory failure with oxygen requirement (aOR 14.64; 95% CI 1.45–147.93), hypertension (aOR 3.14; 95% CI 1.47–6.71), having an arrhythmia (aOR 2.95; 95% CI 1.00–8.68), and obesity (BMI ≥30 kg/m^2^) (aOR 3.35; 95% CI 1.58–7.09) were significantly associated with hospitalization.

**Fig 1:**
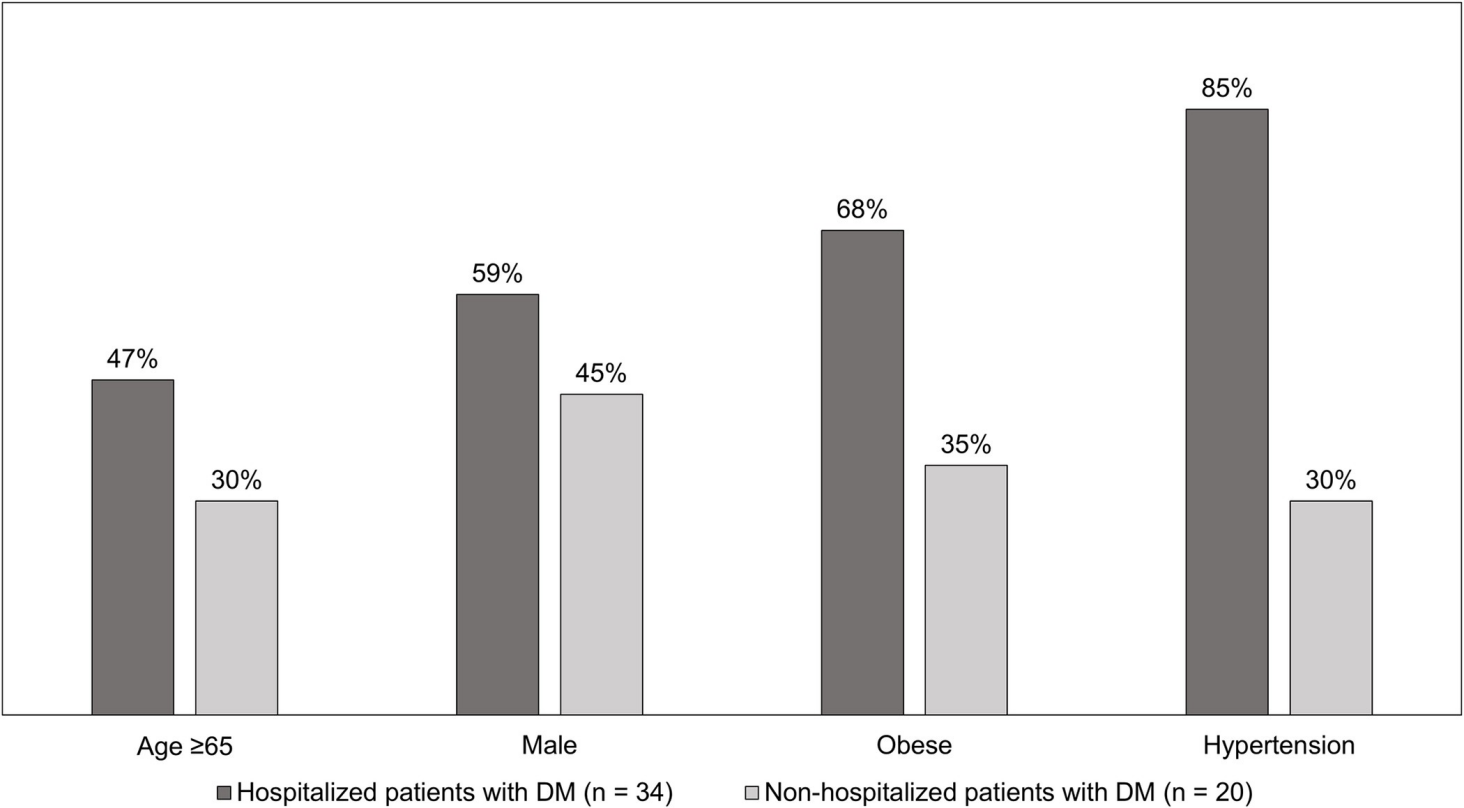
Percentage of select characteristics among laboratory-confirmed COVID-19 patients with diabetes mellitus (DM), by hospitalization status (n = 54)—Colorado, March 2020.

DM was reported as an underlying medical condition for 34 (27%) hospitalized patients and 20 (8%) non-hospitalized patients but was not significantly associated with hospitalization in multivariable analysis. However, when compared to non-hospitalized patients with DM, hospitalized patients with DM were more often ≥65 years old, male, obese, hypertensive, and had at least one other underlying condition adjusted for in our multivariable analysis (32 [94%] versus 11 [55%]) (Fig 1). Finally, patients with metabolic syndrome (the coexistence of DM, hypertension, and obesity) had significantly higher odds of hospitalization in multivariable analysis (aOR 5.71; CI 1.18–27.54).

## Discussion

Among patients with laboratory-confirmed COVID-19 in Colorado, our analysis found many patient characteristics, underlying medical conditions, and medications to be associated with hospitalization in crude analysis. However, after adjusting for previously described risk factors for severe disease, nine factors were found to be associated with hospitalization due to COVID-19, some that have been previously described (i.e., age, sex, obesity, hypertension, arrhythmia, metabolic syndrome) and others appear to be newly identified (i.e., chronic hypoxemic respiratory failure with oxygen requirement and opioid use). In addition, we did not detect a significant association between hospitalization and some factors that have been previously associated with hospitalization or other indicators of severe COVID-19, including race and ethnicity. Some differences in findings of this analysis and previous reports examining risk factors for severe COVID-19 are expected, due to differences in data collection methods (e.g., interview versus medical record abstraction), measures of disease severity used as outcomes (e.g., hospitalization, ICU admission, mechanical ventilation, or death), and how underlying medical conditions are distributed among different populations and categorized in analyses.

Older age and male sex have been consistently identified as risk factors for hospitalization and severe COVID-19 illness in many studies [7, 8, 13, 14, 27, 29, 48]. The increased risk of poor outcomes among older patients is likely, in part, related to waning immune function that comes with aging [49]. The biologic mechanism causing more severe COVID-19 in males compared to females is unknown but likely multifactorial [12]. The finding of older patients, and in some cases older male patients, having more severe disease is similar to many other viral respiratory diseases (e.g., influenza, SARS, MERS, respiratory syncytial virus) [50–52].

There is increasing evidence of the association between obesity and worse outcomes among persons with COVID-19 [7, 13, 27–30, 36–38, 53]. A dose-dependent effect among classes of obesity was present in our univariable analysis, but because of a large amount of overlap in CIs between classes, they were collapsed to a single “obese” category for multivariable analysis. Obesity has been linked to the development of several chronic conditions, including sleep apnea, coronary artery disease, type 2 DM, and hypertension [54]. Once we adjusted for obesity in multivariable analysis, several of these related conditions were no longer significantly associated with hospitalization. Since we also adjusted for age in multivariable analysis, the association between hospitalization and obesity suggests that patients with obesity are more likely to be hospitalized, regardless of age, which has been found by others [32, 36].

While cardiovascular disease, a category inclusive of multiple individual conditions, has been identified as a risk factor for hospitalization and severe COVID-19 [2, 10, 15], it was not statistically significant in our multivariable analysis. Previous reports have found individual cardiac conditions (e.g., congestive heart failure, coronary artery disease, atrial fibrillation/arrythmia, and hypertension) to be associated with hospitalization [7, 26, 27, 33, 41–43, 55]. Within this disease category, we found that history of hypertension or arrhythmia was associated with increased risk of hospitalization in multivariable analysis. However, we did not capture information regarding the severity (e.g., stage) or control of hypertension so we do not know how much of our observed effect might be because of uncontrolled hypertension. Despite an observed higher frequency of hyperlipidemia in hospitalized participants, we did not evaluate its association with hospitalization given the condition was reported in a free text field, reported less frequently during interviews, and many non-hospitalized patients had limited or no medical records available for review. However, in the few studies that systematically collected hyperlipidemia through chart review, patients with hyperlipidemia were less or similarly likely to be hospitalized with COVID-19 [6, 56].

Similar to cardiovascular disease, chronic respiratory disease as a category was not significantly associated with hospitalization in multivariable analysis. There is mixed evidence in the literature regarding chronic respiratory disease as a risk factor for hospitalization and severe COVID-19 [7, 8], which could be related to chronic respiratory disease being a diverse group of conditions that occur in different demographics groups and individual diseases might have different associations with COVID-19 outcomes. For instance, some evaluations found chronic obstructive pulmonary disease (COPD) associated with worse COVID-19 outcomes, while asthma was often found to have no or a protective association with worse COVID-19 outcomes [31, 38, 41, 42]. Although we did not find COPD to be associated with hospitalization, our analysis identified 13/14 individuals with chronic hypoxemic respiratory failure with an oxygen requirement in this investigation were hospitalized. This finding is not surprising but suggests that the severity of underlying medical conditions might further influence their association with COVID-19 hospitalization. While smoking was associated with hospitalization in crude analysis, it was not significant in multivariable analysis. Findings of previous studies of smoking and COVID-19 outcomes have been mixed, including increased, decreased, or no impact on risk as was found in this analysis [16, 39, 57–59]. Smoking has also been found to causes variable risk for SARS-CoV-2 infection suggesting a potential complex affect [60].

In our multivariable analysis, DM was not significantly associated with hospitalization despite being significantly associated in crude analysis. However, when DM was evaluated as part of metabolic syndrome, the syndrome was significantly associated with hospitalization suggesting that DM might only be a risk factor for hospitalization when in combination with other underlying conditions. Metabolic syndrome has been found, in at least one other study, to be associated with multiple negative outcomes among hospitalized COVID-19 patients [61]. We did not differentiate between type 1 and type 2 DM in our analysis, so we are unable to determine if the relationship between DM and COVID-19 hospitalization varies by type. Additionally, we had a lower number of patients with DM, which might have reduced our power to detect an association in multivariable analysis, as was reported in at least one other study [35] compared to studies where DM was reported to occur at a higher frequency and was found to be a risk factor for hospitalization [7, 27, 29, 30, 34, 48, 55, 56]. Compared to other U.S. states, Colorado’s population is relatively healthy having the lowest proportion of adults with obesity and the fourth lowest percentage of residents with at least one of six underlying medical conditions found to be associated with an increased COVID-19 case fatality ratio in China [62, 63].

Race and ethnicity were not found to be significantly associated with hospitalization in this analysis, in contrast to other reports that found persons of Black race [13–15, 26, 29–31, 40, 64] or Hispanic ethnicity [7, 33] had worse COVID-19 outcomes. Overall, we had a small number of Black participants in this analysis, which is consistent with the racial makeup of Colorado [65], and likely substantially limited our ability to identify an association between Black persons and hospitalization. Our cohort had a notable proportion (20%) of participants who identified as Hispanic, which is also consistent with the Colorado population [65]. Of at least five previously published U.S. cohort studies that reported ethnicity, two found an association between persons of Hispanic ethnicity and hospitalization due to COVID-19 [7, 33]. Both studies included >5,000 participants of whom 25–49% were Hispanic compared to the three other studies and our own that had <2,000 participants of whom 15–28% were Hispanic [26, 30, 31]. This suggests that the association between persons of Hispanic ethnicity and hospitalization due to COVID-19 might only be detected in large cohort studies.

One of the unique attributes of this evaluation is the collection of medication and supplement use from patient interviews, which allowed for exploration of the use of prescribed and OTC medications taken in the 30 days prior to COVID-19 symptom onset and their association with hospitalization. It has been hypothesized that patients taking ACE inhibitors or ARBs might be at increased risk for more severe COVID-19 based on SARS-CoV-2 binding to ACE2 receptors found on epithelial cells in the respiratory tract as well as in intestine, kidney, and blood vessels [17, 18, 66, 67]. In our analysis, a larger proportion of hospitalized patients reported taking ACE inhibitors and ARBs than non-hospitalized patients, but the difference was not significant after adjusting for several factors, including age and hypertension, in multivariable analysis. This finding is consistent with several other analyses examining ACE inhibitor and/or ARB use as risk factors for worse COVID-19 outcomes, including at least one that suggests a protective effect against adverse COVID-19 outcomes [21–25, 68]. We also did not detect an association with NSAID use in the 30 days prior to illness onset and hospitalization due to COVID-19, despite previous concerns regarding NSAIDs use and increased risk of worse COVID-19 outcomes [18, 20].

Although no association was seen with ACE inhibitor, ARB, or NSAID use, an association between hospitalization due to COVID-19 and reported opioid use in the 30 days prior to illness onset was observed. Several mechanisms have been proposed for how opioids might cause worse COVID-19 outcomes, including respiratory depression, suppression of immune function, and drug interactions [69]. Given the small number of participants reporting taking these medications and resulting wide confidence intervals, the actual magnitude of the association cannot be predicted with certainty. However, this potential association between COVID-19 and medications that might interfere with respiratory and immune function deserves further evaluation, particularly given the current opioid epidemic in the United States [70].

This investigation is subject to multiple limitations. First, interviews were conducted several weeks after illness onset, which allowed for accurate classification of patients by hospitalization status but might have led to recall bias. However, time between illness onset and interview did not differ by hospitalization status so any existing recall bias should not significantly influence associations with hospitalization. Second, non-hospitalized participants were less likely to have medical records related to their COVID-19 illness available for abstraction (n = 88 or 37% of non-hospitalized patients had records available for review) biasing the number of underlying medical conditions reported towards those who were hospitalized. Third, BMI was calculated using self-reported height and weight. Given the tendency for people to under-report their weight, and the higher degree of under-reporting among those in higher BMI categories, the actual association between obesity and severe disease might be underestimated [71]. Fourth, our findings are specific to this population and might not be generalizable to all populations due to the evolution of testing practices and characteristics of infected persons during the pandemic, socioeconomic status, or underlying health status of participants [42]. Fifth, because many demographic or social characteristics, specific underlying medical conditions, and medications were reported by a small number of participants, our ability to make conclusions about their associations with hospitalization was limited and confidence intervals for some estimates were wide. This also prevented adequate exploration and adjustment for interaction between potential risk factors. Lastly, because data were partly self-reported, there is a possibility of response bias.

In this analysis, age ≥65 years, male sex, obesity, hypertension, chronic hypoxemic respiratory failure with an oxygen requirement, arrhythmia, metabolic syndrome, and opioid use were determined to be independent risk factors for hospitalization among COVID-19 patients in Colorado. Understanding risk factors for hospitalization can inform strategic planning and resource allocation at multiple levels including prevention (e.g., vaccine allocation), diagnosis, and treatment. Given the unique findings of this analysis as well as conflicting findings among published studies, further analyses with larger sample sizes of persons from diverse backgrounds throughout the U.S. and worldwide could help build consensus in our understanding of what patient characteristics, medications, and underlying medical conditions are associated with hospitalization and worse outcomes in persons with COVID-19. Persons in high risk groups should be targeted for tailored public health messaging, prioritized for preventive measures, and should receive appropriate clinical management as soon as possible after developing symptoms compatible with COVID-19. It is important to remember that all persons, regardless of demographics, medication use, and underlying medical conditions are at risk for severe COVID-19 illness and should take all recommended precautions to prevent infection and transmission including mask wearing, social distancing, and hand hygiene.
